# Cortisol and inflammatory biomarker levels in youths with attention deficit hyperactivity disorder (ADHD): evidence from a systematic review with meta-analysis

**DOI:** 10.1038/s41398-021-01550-0

**Published:** 2021-08-19

**Authors:** Jane Pei-Chen Chang, Kuan-Pin Su, Valeria Mondelli, Carmine M. Pariante

**Affiliations:** 1 Department of Psychological Medicine, Institute of Psychiatry, Psychology and Neuroscience, King’s College London, London, UK; 2 Department of Psychiatry and MBI-Lab, China Medical University Hospital, Taichung, Taiwan; 3 College of Medicine, China Medical University, Taichung, Taiwan; 4 An-Nan Hospital, China Medical University, Tainan, Taiwan

**Keywords:** Neuroscience, Physiology

## Abstract

Several studies reported abnormal cortisol and inflammatory biomarker levels in youths with attention deficit hyperactivity disorder (ADHD), but the results have not been conclusive. We conducted a systematic review followed by a meta-analysis of case-control studies assessing blood or saliva cortisol levels and blood levels of inflammatory biomarkers in youth with ADHD. The effect sizes (ES) were synthesized by using a random-effects model. In the 19 studies on cortisol levels (totaling *n* = 916 youth with ADHD and *n* = 947 typically developing (TD), healthy youth), youth with ADHD have lower basal cortisol levels at any time-points during the day (effect size: .68; *p* = 0.004) and lower cumulative levels of cortisol (ES: .39, *p* = .008) throughout the day than TD youth. Moreover, morning cortisol levels were lower in ADHD youth when compared with TD youth (14 studies, *n* = 1679, ES: .84, *p* = 0.003), while there is no difference for the afternoon cortisol levels (*p* = 0.48). The meta-analysis on inflammation biomarker was conducted on 4 studies (totaling *n* = 404 youth) showed that Tumour Necrosis Factor-alpha (TNF-α) was lower in ADHD when compared with TD (3 studies, *n* = 257 youth, *p* = 0.004), while no differences for Interleukin-1β(IL-1β) (*p* = 0.21), IL-6 (*p* = 0.09) and IL-10 (*p* = 0.77). The lower cortisol in the context of low TNF-α levels may indicate a specific pattern of biomarkers in ADHD, and further investigation is warranted.

## Introduction

Hypothalamus-Pituitary-Adrenal (HPA) axis dysfunction and inflammation have been suggested to contribute to the development of attention deficit hyperactivity disorder (ADHD). Steingard et al. were the first to describe abnormalities of the HPA axis in children with ADHD, as they described a higher rate of dexamethasone non suppression in children with ADHD when compared with controls (22.7% vs. 0%) [1]. Subsequently, Kaneko et al. [2]. found that only around 40% of children with ADHD had normal diurnal cortisol rhythm, defined as a diurnal cortisol variation showing a maximum level in the morning and a minimum level at night, as compared with 90% of adult controls and 81% of children with autistic spectrum disorders; moreover, abnormal cortisol rhythms were more frequently found in children with severe ADHD compared with children with mild ADHD [2]. However, subsequent studies have found less consistent findings. For example, one study comparing ADHD and typically developing (TD) children reported a lower morning salivary cortisol level in children with ADHD [3], while another study reported no differences [4]. Moreover, while some studies demonstrated a correlation between low basal cortisol levels and hyperactivity [2, 5] and total ADHD symptoms [5], other studies failed to report such association [6]. There had only been two systematic reviews and meta-analyses of biomarkers, including cortisol, in both children and adults with ADHD: one did not perform subanalysis in regards to the timing of sample collection [7], and the other only included adult subjects [8].

Inflammation has also been suggested to play a crucial role in the pathogenesis of ADHD. Epidemiology, genetic studies, and a recent systematic review have provided support by showing high comorbidity of ADHD with inflammatory and autoimmune disorders and the differences in biomarkers between ADHD and TD youth [9–13]. For example, ADHD is more likely to have allergy associated disorders including asthma, rhinitis, atopic dermatitis and allergic conjunctivitis [9], and psoriasis [10]. Moreover, a personal and maternal history of autoimmune diseases, such as thyrotoxicosis, type 1 diabetes, autoimmune hepatitis, psoriasis, and ankylosing spondylitis, has been associated with an increased risk for ADHD [11].

Furthermore, ADHD has been suggested as the result of an exaggerated central nervous system (CNS) inflammatory response in fetus associated with maternal inflammation [14]. Neonatal infection associated with inflammatory responses and systematic inflammation during the first postnatal month has been shown to be associated with the risk for ADHD at 10 years of age [15]. As for the genetic studies, De Jong et al. [12]. found a similar genetic signature between ADHD and depression in genes related to inflammation. On the other hand, the studies regarding inflammatory biomarkers in ADHD have been inconclusive. For example, some studies reported a higher immunoreactivity and higher levels of a pro-inflammatory cytokine such as IL-6 and anti-inflammatory cytokine such as IL-10 in ADHD children [16, 17], while others found no differences in IL-1b, IL-6, IL-10 levels between ADHD and normal control adults [18, 19]. In addition, there has only been one study reporting higher c-reactive protein (CRP) levels in children with ADHD when compared with TD children [20], while another study reported no association between ADHD symptoms and CRP in a non-ADHD sample [21].

In this study, we have updated the previously published meta-analysis [7] of cortisol levels by including seven recent studies [3, 20, 22–26]. Noteworthy, we assessed also the time of sample collection in potentially explaining the variability in the findings, not considered by the previous meta-analysis [7]. Moreover, to our knowledge there has been no meta-analysis on the inflammatory biomarkers in children with ADHD. Thus, our study also provides the first meta-analystic findings by pulling together all the cross-sectional studies on inflammatory biomarkers in children with ADHD.

## Methods

We conducted a systematic review and a meta-analysis in accordance with the Preferred Reporting Items for Systematic Reviews and Meta-Analysis (PRISMA) guidelines [27].

### Literature search

To identify eligible studies in the systematic review and the meta-analysis, a computerized search was performed for studies available as of 19th of January 2021 in the electronic databases of PubMed at the National Library of Medicine. The search for cortisol levels was performed by using the search terms: (attention deficit hyperactivity disorder OR ADHD) AND cortisol, without special limitation in language. References lists from identified articles and relevant reviews were scrutinized for studies not indexed in the electronic databases. The search for inflammatory biomarkers was performed by using the search terms: (attention deficit hyperactivity disorder OR ADHD) AND inflammation; (attention deficit hyperactivity disorder OR ADHD) AND (c-reactive protein or CRP); (attention deficit hyperactivity disorder OR ADHD) AND interleukin; (attention deficit hyperactivity disorder OR ADHD) AND (tumour necrosis factor-α or TNF-α), without special limitation in language. References lists from identified articles and relevant reviews were scrutinized for studies not indexed in the electronic databases. Our initial search identified 154 studies for the cortisol levels (see Supplementary Fig. S1) and 273 studies for the inflammatory biomarker levels (see Supplementary Fig. S2).

### Inclusion criteria of studies in the meta-analysis

The characteristics and references of the included articles are described in Tables 1 and 2.

**Table 1:** Characteristics of Studies Included in the Meta-Analysis for Cortisol Levels in Children with ADHD.

Studies	ADHD, *n* (male, %)	TD, *n* (male, %)	Med (%)*	Age (yrs), mean (SD), or age range	Country	Sample Origin, Time
Jansen (1999) [79]	10 (100)	15 (86.7)	NS	ADHD: 9.8 (1.5) TD: 10 (2.0)	NL	S, PM
Snoek (2004) [80]	23 (82.6)	26 (76.9)	73.9	ADHD: 9.8 (1.4) TD: 10.3 (1.3)	NL	S, PM
White (2005) [81]	12 (83)	21 (79)	0	ADHD: 5–12 TD: 6–13	USA	S, PM
Blomqvist (2007) [82]	18 (83.3)	71 (66.2)	16.7	13	Sweden	S, AM PM
Randazzo (2008) [83]	13 (61.5)	19 (31.6)	0	Boys: 9–13 Girls: 8–12	USA	S, PM
Freitag (2009) [84]	52 (79)	69 (48)	37^$^	ADHD: 9.4 (1.7) TD: 10.0 (1.5)	Germany	S, AM
Maldonado (2009) [85]	33 (45.5)	33 (57.6)	0	ADHD: 6.5 (.15) TD: 6.2 (.13)	Spain	S, AM
Van West (2009) [86]	52 (86.5)	25 (80)	0	ADHD: 8.5 (1.8) TD: 8.9 (1.5)	Belgium	S, PM
Christiansen (2010) [87]	62 (NS)	61 (NS)	37.1	ADHD: 10.6 (2.8) TD: 10.5 (2.7)	Germany	S, AM
Ma (2011) [88]	128 (100)	30 (100)	NS	ADHD: 9.6 (2.4) TD:10.2 (3.3)	China	B, AM
McCarthy (2011) [89]	28 (66)	334 (48)	75.9	4–10	USA	S, AM
Wang (2011) [90]	50 (80)	50 (80)	0	ADHD: 7.6 (1.6) TD:7.8 (1.5)	Taiwan	S, AM
Imeraj (2012) [91]	11 (82)	33 (79)	0^@^	ADHD: 8.8 (1.5) TD: 8.9 (1.6)	Belgium	S, D
Isaksson (2012) [92]	161 (75.6)	168 (41.6)	89	6–17	Sweden	S, D
Kuppili (2017) [93]	30 (93)	30 (93)	0	ADHD: 9.47 (2.43) TD: 10.30 (2.79)	India	B, AM
Angeli (2018) [94]	42 (78.6)	40 (62.5)	0	ADHD: 8.4 (1.9) TD:8.1 (1.7)	Greece	S, D
Isik (2018) [95]	77 (74)	42 (69)	0	ADHD: 10.2 (2.1) TD: 10.9 (2.8)	Turkey	B, AM
Anesiadou (2021) [96]	34 (65)	24 (67)	0	ADHD: 8.79 (1.43) TD: 9.74 (1.98)	Greece	S,D
Chang (2020) [97]	95 (86)	21 (71)	0	ADHD: 9.32 (3.05) TD: 9.19 (2.96)	Taiwan	S, D

Note, *ADHD* attention deficit hyperactivity disorder, *AM* morning, *B* blood, *D* diurnal, *N* number, *NL* Netherlands, *NS* not specified, *PM* afternoon, *S* saliva, *SD* standard deviation, *TD* typically developing youth, *yrs* years.

*Indicates % of ADHD medication use in ADHD group; ^$^Indicates no meds prior to sampling; ^@^Indicates no meds 72 h prior to sampling.

**Table 2:** Characteristics of Studies Included in the Meta-Analysis for Inflammatory Biomarker Levels in Children with ADHD.

Studies	ADHD, *n* (male, %)	TD, *n* (male, %)	Med (%)^a^	Age (yrs), mean (SD), or age range	Country
Oades (2010) [98]	21 (67)	21 (95)	0	ADHD: 9.84 (1.4) TD: 11.0 (1.5)	Germany
Verlaet (2019) [99]	57 (71)	69 (65)	0	ADHD: 8.98 (1.75) TD: 8.37 (1.69)	NL
Darwish (2019) [100]	60 (83)	60 (68)	0	ADHD: 8.4 (1.28) TD: 8.73 (1.89)	Egypt
Chang (2020) [101]	95 (86)	21 (71)	0	ADHD: 9.32 (3.05) TD: 9.19 (2.96)	Taiwan

Note *ADHD* attention deficit hyperactivity disorder, *N* number, *NL* Netherlands, *SD* standard deviation, *TD* typically developing youth, *yrs* years.

^a^Indicates % of ADHD medication use in ADHD group.

#### ADHD and cortisol levels

Our criteria were: the studies (1) measured levels of cortisol of blood or salivary sample; (2) participants were school-aged children (4–12 years) and adolescents (13–17 years) who had a diagnosis of ADHD; (3) the data allowed to calculate an effect size; and (4) the publications were in peer-reviewed journals. Nineteen studies [3–5, 20, 22–26, 28–37] were included in the meta-analysis on cortisol levels.

#### ADHD and Inflammatory Biomarker Levels

Our criteria were: the studies (1) measured levels of inflammatory biomarkers including C-reactive protein, interleukin (IL)−1β, IL-6, IL-10, and tumour necrosis factor (TNF-)α; (2) participants were school-aged children (4–12 years) and adolescents (13–17 years) who had a diagnosis of ADHD; 3) the data allowed to calculate an effect size; and (4) the publications were in peer-reviewed journals. Four studies [19, 20, 38, 39] were included in the meta-analysis on inflammatory biomarker levels.

Studies that included and re-analysed the same data set as previously published studies were not regarded as independent, and in this case only the study with the highest number of participants was included. See Supplementary Fig. S1 and Supplementary Fig. S2 for the flowchart showing the selection of included studies. To assess the quality of the included cross-sectional studies, we used the Joanna Briggs Institute (JBI) Critical Appraisal Checklist for Analytical Cross-Sectional Studies [40]. This tool considers the following: (1) definition of the inclusion criteria,(2) description of the study subjects and the settings, (3) valid and reliable measurements of the exposure, (4) objective and standard criteria for measurement, (5) confounding factors, (6) strategies for confounding factors, (7) valid and reliable measures of outcomes, and (8) statistical analysis used. The results of the appraisal were used to inform the synthesis and interpretation of the review results. See Supplementary Tables S1 and S2 for the quality check of the included studies.

### Meta-analytic methods

In our analysis, the primary outcomes were comparisons of (1) cortisol levels and (2) levels of IL-1β, IL-6, IL-10, and TN-α, between ADHD and controls. For each identified study, the effect size (*ES*) expressing the difference in the cortisol levels ((or the area-under-the-curve with respect to the ground (AUCg) if diurnal cortisol was measured in the study) or differences in the inflammatory biomarker levels between ADHD and controls, were described as the standardized mean difference (SMD) on the basis of Hedge’s adjusted *g*, in which a value lesser than 0 indicated that cortisol levels or inflammatory biomarker levels were higher in ADHD subjects. When these data could not be retrieved from the publications, we contacted the authors to acquire the data of derived *ES* from other measures of variability. The results of individual studies were synthesized by the random-effects model [41], by which ESs were pooled and 95% confidence intervals (CIs) were calculated. The significance of the pooled effect size was determined by the z test. Sensitivity analyses were performed to determine whether any individual study was responsible for the significant results; moreover, each study was individually removed and the significance was retested. The main results of the meta-analysis did not change after the removal of any one of the included studies. The *I*^*2*^ statistic assessed heterogeneity between studies. Publication bias was assessed using the Egger regression asymmetry tests (and inspection of the regression asymmetry plot) and the Begg adjusted rank correlation test. There was no publication bias in the 19 studies (Begg’s test, *p* for bias = 0.916; Egger’s test, *p* for bias = 0.434). Meta-analyses were conducted by applying STATA [42] and Forest Plots were created by using Review Manager 5.3 [43]. Two-sided *p* values < 0.05 were considered statistically significant.

## Results

### Youth with ADHD have lower cortisol levels

**Fig. 1:**
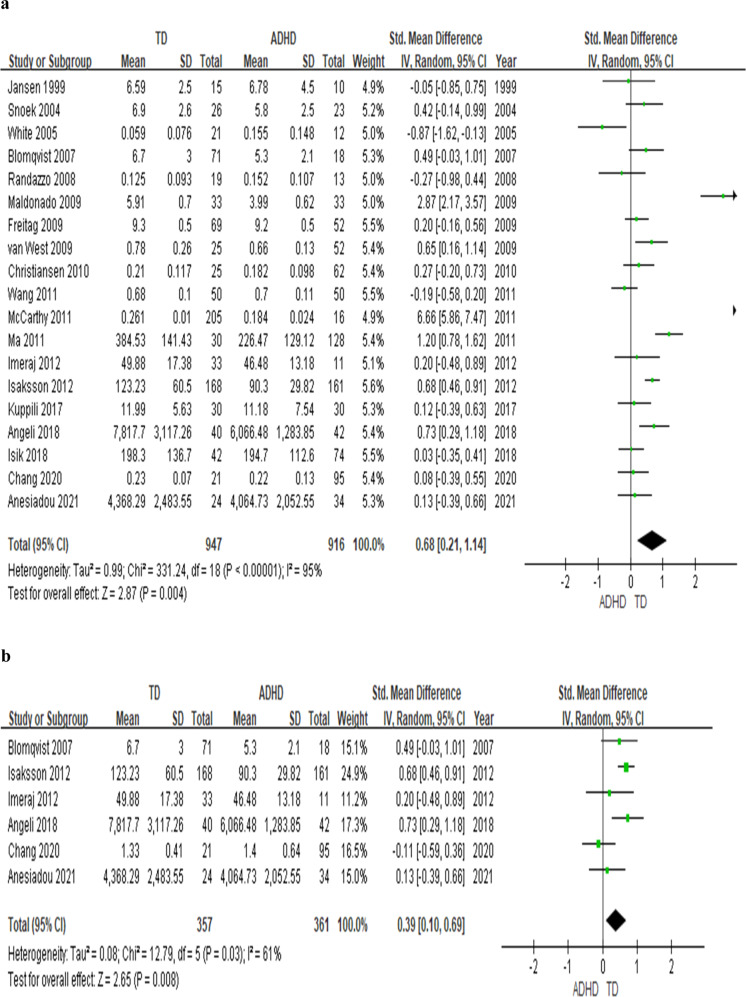
Forest plots comparing effect sizes of basal cortisol levels and AUCg between ADHD and TD groups. Forest plots showing effect sizes (Hedges’s g) and 95% confidence intervals (CIs) from individual studies and pooled results comparing (**a**) cortisol levels at any time point throughout the day and (**b**) cortisol AUCg between ADHD population and TD group. Note, ADHD attention deficit hyperactivity disorder, AUCg area-under-the-curve with respect to the ground, CI confidence interval, Std standard, TD typically developing youth.

**Fig. 2:**
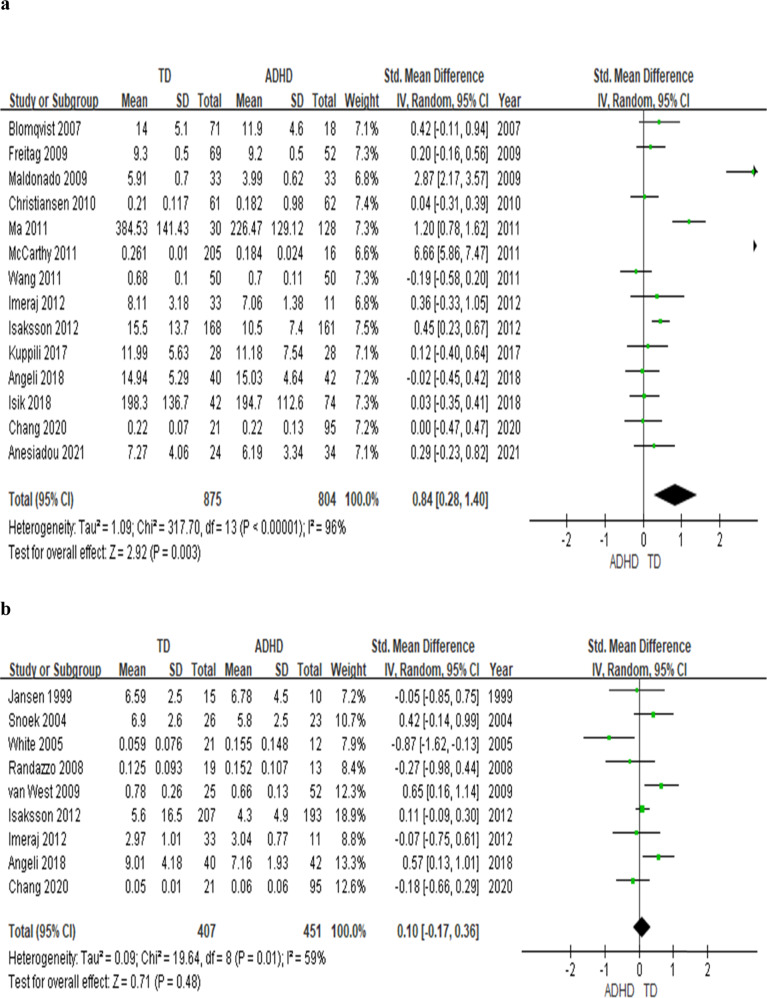
Forest plots comparing effect sizes of morning and afternoon cortisol levels between ADHD and TD groups. Forest plots showing effect sizes (Hedges’s g) and 95% confidence intervals (CIs) from individual studies and pooled results comparing (**a**) morning and (**b**) afternoon cortisol levels between ADHD population and TD group. Note, ADHD attention deficit hyperactivity disorder, CI confidence interval, Std standard, TD typically developing youth.

The meta-analysis on 19 studies comparing basal cortisol levels (saliva and blood) in children with ADHD and TD children showed that ADHD youth have lower basal cortisol levels, independently from the time-point (*n* = 1863, *g* = 0.68, *p* = 0.004) (see Fig. 1a) and lower AUCg (*n* = 718, *g* = 0.39, *p* = 0.008) (see Fig. 1b). In addition, when we analysed the 14 studies that measured the morning cortisol levels, youth with ADHD continue to have a lower cortisol level than TD youth (*n* = 1679 participants, *g* = 0.84, *p* = 0.003) (see Fig. 2a). On the other hand, the subanalysis of 9 studies with the afternoon salivary cortisol levels showed no difference between ADHD and TD groups (*n* = 858, *p* = 0.48) (see Fig. 2b).

Since salivary sampling is a non-invasive and convenient method to measure cortisol, thus we repeated the analysis after exclusion of three studies that measured blood cortisol (both in the morning), and found again that youth with ADHD have lower salivary cortisol levels than TD youth (*n* = 1570 *g* = 0.80, *p* = 0.006) (see Supplementary Figure S3a). The subanalysis of 11 studies measuring morning salivary cortisol levels (*n* = 1354, *g* = 0.99, *p* = 0.006) and 6 studies measuring awakening salivary cortical levels (*n* = 750, *g* = 0.27, *p* = 0.002) confirmed that youth with ADHD have lower salivary cortisol levels than TD youth (Supplementary Fig. S3b and c). The subanalysis of 3 studies measuring noon salivary cortisol levels (*n* = 242, *p* = 0.07) (Supplementary Fig. S4a) and the subanalysis of 5 studies measuring bedtime salivary cortisol levels (*n* = 704, *p* = 0.51) showed no differences between youth with ADHD and TD youth (see Supplementary Fig. S4b).

### Youth with ADHD have lower TNF-α levels

**Fig. 3:**
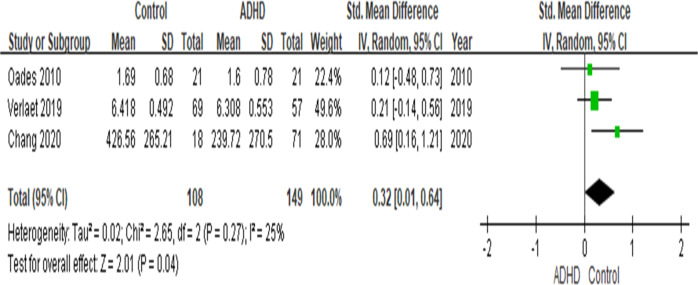
Forest plots comparing effect sizes of TNF-α levels between ADHD and TD groups. Forest plots showing effect sizes (Hedges’s g) and 95% confidence intervals (CIs) from individual studies and pooled results comparing TNF-α levels between ADHD population and TD group. Note, ADHD, attention deficit hyperactivity disorder, CI confidence interval, Std standard, TD typically developing youth, TNF-α tumour necrosis factor-alpha.

The meta-analysis on 3 studies comparing TNF-α levels in children with ADHD and TD children showed that ADHD youth have lower TNF-α levels *(n* = 257, *g* = 0.32, *p* = 0.004) (see Fig. 3). However, there were no differences in IL-1β (*p* = 0.21) or IL-10 (*p* = 0.77) levels between children with ADHD and TD children (see Supplementary Fig. S5a and c). However, there is a trend showing that ADHD youth have higher IL-6 levels than TD youth (*p* = 0.09) (see Supplementary Fig. S5b).

## Discussion

The major findings of our meta-analyses are that ADHD youth have lower basal cortisol levels at any time point, lower AUCg, lower morning cortisol levels, and lower TNF-α levels when compared with TD youth. To our knowledge, this is the first meta-analysis of cortisol levels of children with ADHD to include cortisol samples from both blood and saliva and examining sample collection time as a variable, and also the first meta-analysis of inflammatory biomarker levels of children with ADHD.

Morning salivary cortisol level seems to be a potential biomarker for children with ADHD, since it provides a non-invasive alternative to serum cortisol levels which closely correlates with plasma-free cortisol [44]. Overall, our results showing a lower morning cortisol level, especially awakening cortisol level, in ADHD youth may provide a partial explanation of why youth with ADHD may feel tired and have a later rise time in the morning [45], since their cortisol levels may require a longer time to peak. An altered diurnal rhythm of cortisol levels may contribute to the low morning cortisol levels in children with ADHD. A phase delay of cortisol rhythms in ADHD has been further supported by a study of adults with ADHD [46], where adults with ADHD had a delayed phase of cortisol rhythm by 2 h. Incidentally, the lack of difference in the afternoon salivary cortisol levels between youth with ADHD and TD youth may be explained by the higher degree of exposure to stressors, including familial conflicts, in the youth with ADHD throughout the day [47]. For example, children with ADHD often have poor peer and family interactions and encounter stressful scenarios at school and at home [47, 48], which may cause a continuous increase in free cortisol levels. This evidence points at the HPA axis as a potential therapeutic target for ADHD. Medications for ADHD, such as methylphenidate, are able to increase cortisol levels by triggering dopamine release in the central nervous system [49], while glucocorticoids administration improves impulse control in continuous performance tests [50], possibly by enhancing the effects of dopamine in the meso-limbic system [51]. Furthermore, treatment with stimulant medication has been shown to increase baseline cortisol levels [52].

In addition, many other factors may also affect the cortisol levels, such as length of rest period before sample collection, chronic use of nicotine, alcohol, medications, common heterozygous mutations of the 21-hydroxylase gene (a decreased adrenal 21-hydroxylase activity will interfere with cortisol biosynthesis) [53]. However, these factors are often less mentioned in most of the studies. We have carried out a qualitative examination on the aforementioned factors in the included studies of our meta-analysis (see Table 1 and Supplementary Table S3) and found that although most of the studies [3–5, 20, 22–26, 29–34, 36, 37] reported ADHD medication use status except for Jansen et al. [28] and Ma et al. [35], none mentioned about the 21-hydroxylase genetic mutations. In addition, although most of the included studies, other than those collected awakening salivary cortisol levels [3, 20, 22, 23, 26, 32], did not report the resting time required prior to the sample collection, however, they did ask the patients to have at least 30 min to a 90 min gap between brushing their teeth, eating/ drinking and the sample collection; [4, 5, 20, 24, 25, 31, 34–37] and at least a 90 min to a 24 h restriction of strenuous exercises prior to the sample collection [5, 20, 25, 35]. What is more striking is that only 3 studies [5, 20, 25] asked their participants not to drink alcohol or smoke prior to the sample collection or mentioned substance use disorder in the exclusion criteria, although about more than half the studies [3, 20, 24, 25, 30–32, 34, 35, 37] included in the meta-analysis enrolled teenagers with age ≥13, a population relatively at risk for substance use. Therefore, factors such as chronic nicotine or alcohol use and genetic mutation of the 21 hydroxylases should be examined in detail in future studies studying cortisol levels in youth.

Although the sensitivity analysis showed that the main results of the meta-analysis did not change after the removal of any one of the included studies. Maldonado et al. 2009 [33] and McCarthy et al. 2011 [36] seem to drive the effects in the basal cortisol (Fig. 1a) and morning cortisol analyses (Fig. 2a). Thus, we performed a subanalysis excluding the two studies and found the results for the basal cortisol levels and the morning cortisol levels remained significant (*g* = 0.27; *p* = 0.010; *g* = 0.24; *p* = 0.020, respectively). The heterogeneity (I^2^) also decreased (for the basal cortisol levels I^2^ decreased from 95% to 73.1%; for the morning cortisol levels, I^2^ decreased from 96–65.5%). These two studies may differ from the rest of the included studies in that they included participants much younger than the rest of the other studies (most of the studies included participants older than 6 years of age), where the participants in Maldonado et al.’s study range from 5–8 years of age and in McCarthy et al.’s study range from 4–10 years of age. Moreover, the inclusion criteria for McCarthy’s study are parental reports of the ADHD diagnosis and the use of stimulants, rather than diagnosis made by clinical professionals or by DSM structured interviews.

Of note, it has been reported that the immunoassays measuring cortisol do not just measure cortisol, but also measure inactive 5α-reduced metabolites of cortisol [54, 55]. Therefore, it may not be low levels of cortisol but rather low levels of the non-active 5α-reduced metabolite of cortisol associated with psychiatric disorders or the severity of the psychiatric disorders [54, 55]. Thus, in order to elucidate the relationship of cortisol in youth with ADHD, the use of mass spectrometry may be necessary to accurately understand the role of cortisol in ADHD.

Our study finding is overall consistent with Scassellati’s meta-analysis of 8 studies of salivary cortisol levels, where patients with ADHD had lower cortisol levels when compared to TD subjects (*p* = 0.0001) [7]. We have updated this meta-analysis by adding five more recent studies, measuring salivary cortisol levels [3, 20, 22, 23, 26], and also by including three other studies [30, 31, 36], not included by Scassellati et al. meta-analysis. It is also of note that Bonvicini et al.’s meta-analysis of 3 studies of adults with ADHD (*n* = 117) showed no difference between salivary cortisol levels of ADHD and normal controls (*p* = 0.13), but there was a numerical trend showing adults with ADHD having lower salivary cortisol levels [8].

Another interesting finding of our meta-analysis is that children with ADHD have a lower level of TNF-α than control children. This finding is different from previous study findings showing no difference in TNF-α levels in ADHD subjects, both youth and adults, and normal controls [18, 19]. On the other hand, lower levels of TNF-α have been positively associated with reaction time (RT) variability in the Continuous Performance Test (indicating inattention) in ADHD [56]. RT variability is the core feature of the hypothesis of glial impairment in ADHD [57] and has been suggested as an endophenotype for ADHD [56]. Moreover, a study addressing the association between ADHD and polymorphism of TNF-α genes in children with ADHD and healthy controls showed that the 308 polymorphism (alleles 1 and 2) at the TNF-α gene is correlated with two attention measures, the hand-eye test of the Neurobehavioral Evaluation System (NES2), which evaluates visual-motor coordination, and sub-test of Shape Discrimination Test (TDTP) [58]. Specifically, individuals without any TNF-α gene allele 2 perform significantly better in the accuracy sub-test of TDTP than individuals with allele 2 (homozygotes or heterozygotes). In addition, a negative correlation has been shown between cortisol awakening response and TNF-α in adults with inattentive type ADHD but not in adults with other subtypes [18]. It has been reported that an imbalance of TNF-α levels, whether too much or too little, can impair cognitive function [59, 60], and the variation of the TNF-α levels may influence the turnover of the monoamines [61]. Thus, inadequate or excess levels of TNF-α may be associated with impaired cognitive performance. More studies with a larger sample will be needed to further clarify the role of TNF-α in ADHD.

Although there is no difference in the interleukin levels between ADHD and TD youth in our meta-analysis, there is a trend showing that IL-6 is higher in ADHD youth than in TD youth. The finding may be not significant due to a lack of power, as genetic, animal, and clinical studies have suggested a potential role of IL-6 in ADHD pathogenesis. For example, one study showed an increased frequency of the C allele at the 174 polymorphism of the *IL-6* gene in children with ADHD; moreover, the A and G alleles of the 174 polymorphism have been associated with measures of attention, with people with the *IL-6* AA homozygotes performing better than *IL-6* homozygotes [58]. Animal studies show that administration of IL-6 in rodent models will alter neurotransmission changes that are similar to the changes seen in ADHD, such as reduced dopamine levels [62]. Moreover, children born prematurely, and with a current or persistent elevation of inflammatory markers, including IL-6, during the first two postnatal weeks, show attention problems at 2 years of age [63]. In addition, a study has shown the decrease in cytokine levels such as IL-6 and CRP, after 8-week omega-3 supplementation, in children with ADHD was correlated with significant improvement in clinical symptoms [64], although our own study did not replicate these findings, and pointed to the need of dividing ADHD children in subgroups based on their biomarker profile [65]. Finally, our systematic review only identified one cross-sectional study comparing CRP levels between ADHD youth and TD youth, where ADHD youth had higher levels of CRP [20]. Thus, more studies on CRP in ADHD are warranted for further investigation. Of note, the situation with high IL-6 and low TNF-α observed in ADHD youth in our study has also been observed in patients with Schizophrenia during both exacerbation and remission [66]. The increase in IL-6 may be associated with the decrease of TNF-α, since IL-6 has been shown to have a suppressive effect on TNF-α production [67], and that they mutually counter-regulate each other during the initial phase of an immune response [68].

A number of biological mechanisms have been proposed to explain the HPA axis and inflammatory abnormalities in ADHD. Youth with ADHD may have an under-active behavioural inhibition system (BIS) [69], and an under-active BIS is closely associated with both lower cortisol levels and deficits in working memory, self-regulation of affect, internalization of speech, and reconstitution of goal-directed behavior in ADHD [70]. ADHD has also been associated with psychosocial adversity and prenatal stress [71], which in turn may lead to low cortisol levels [72, 73] and to hyporeactivity of the HPA axis as one way to adapt to chronic stress [72]. Moreover, youth with ADHD often have a lack of physiological arousal when facing challenges, and this low level of physiological arousal may then drive the child to search for sensations that could eventually trigger a decrease in cortisol response to stressors, after repeated activations [74]. Some evidence suggests a common pathway for the development of both HPA axis and inflammatory abnormalities. ADHD often co-occurs with allergic diseases, and some studies have indicated an association between ADHD and streptococcus-mediated neuropsychiatric disorders [75]. It is possible that a sustained and exaggerated release of inflammatory cytokines in atopic eczema (AE) affected children may impact ADHD relevant brain circuits and affecting behaviour and motor control, emotional regulation, or motivational mechanisms [75]. Moreover, ADHD has been shown to have a higher comorbidity with T-cell mediated neuroinflammation [76, 77], such as celiac diseases and atopic diseases.

The main limitation of our meta-analysis is that we did not control for confounders such as diet and exercise, and that we did not subgroup the subjects into the three ADHD subtypes (inattentive, the hyperactive-impulsive, and the combined subtypes), as these data were not consistently present in the included papers. Moreover, we did not account for the cortisol awakening response, therefore we cannot draw any conclusion on the stress reactivity in ADHD. However, a previous meta-analysis on cortisol reactivity in response to a stressor in ADHD showed no difference between cortisol reactivity and ADHD, but implied that there is significant heterogeneity in the analyses that might serve as moderators of this association [78]. In addition, the heterogeneity of the studies should be considered when interpreting the results of our meta-analyses. For example, as mentioned earlier in the discussion section, when we excluded Maldonado et al. and McCarthy et al. from the analyses, the heterogeneity decreased, which may be attributed to the inclusion of a younger age group (<6 years old) and less rigorous inclusion criteria for the diagnosis of ADHD (self-reports vs structured interviews, in the case of McCarthy et al.’s study). It is also important to point out that there is a limited literature [19, 20, 39] (3 studies) on TNF-α in ADHD, thus more studies in the future are warranted to support the potential role of TNF-α as a biomarker in ADHD.

## Conclusion

We find, in our meta-analysis of existing studies, that children with ADHD have lower basal cortisol levels, especially morning cortisol levels, than TD youth. The lower cortisol levels in ADHD further imply the role of HPA axis hypoactivity in ADHD pathogenesis. In addition, since salivary cortisol levels are often normalized after the child receives treatments with stimulant medications; further attention is warranted for the development of potential treatment strategies involving the normalization of salivary cortisol levels in youth with ADHD. Moreover, we also found that ADHD youth have lower TNF-α levels than TD youth, thus suggesting future studies should investigate TNF-α as a potential biomarker in ADHD.

## Supplementary information

Figure S1

Figure S2

Figure S3

Figure S4

Figure S5

Table S1

Table S2

Table S3
