# Supplementary material for: Cortisol and inflammatory biomarker levels in youths with attention deficit hyperactivity disorder (ADHD): evidence from a systematic review with meta-analysis
Source: Transl Psychiatry. 2021 Aug 19;11:430. doi: 10.1038/s41398-021-01550-0 (PMC8377148; doi:10.1038/s41398-021-01550-0)
Supplement: Supplementary file 1 — Figure S1 [file 41398_2021_1550_MOESM1_ESM.doc]

**Screening**

**Included**

**Eligibility**

**Identification**

Records identified through database searching

Records after duplicates removed

Records screened

Records excluded

Full-text articles assessed for eligibility

Full-text articles excluded, with reasons

(n=154)

(n=148)

(n= 90)

(n=58)

(n=90)

(n= 71)

Unsuitable population

(n= 67)

Unavailable data

(n=4)

(n= 19)

Morning (n=14)

Noon (n=3)

Afternoon (n=9)

Bedtime (n=5)

Studies included in quantitative synthesis (meta-analysis)

Supplementary Figure 1. PRISMA Flow Diagram for Cortisol Levels.
