# Supplementary material for: Cortisol and inflammatory biomarker levels in youths with attention deficit hyperactivity disorder (ADHD): evidence from a systematic review with meta-analysis
Source: Transl Psychiatry. 2021 Aug 19;11:430. doi: 10.1038/s41398-021-01550-0 (PMC8377148; doi:10.1038/s41398-021-01550-0)
Supplement: Supplementary file 2 — Figure S2 [file 41398_2021_1550_MOESM2_ESM.doc]

**Screening**

**Included**

**Eligibility**

**Identification**

Records identified through database searching

(273)

Records after duplicates removed

Records screened

Records excluded

Full-text articles assessed for eligibility

Full-text articles excluded, with reasons

Interleukin

(n=45)

(n=230)

(n= 230)

(n=150)

(n=80)

(n=76)

Unsuitable population

(n= 71)

Unavailable data

(n=5)

(n= 4)

IL-1β (n=3)

IL-6 (n=3)

IL-10 (n=3)

TNFα (n=4)

Studies included in quantitative synthesis (meta-analysis)

Supplementary Figure 2. PRISMA Flow Diagram for Inflammatory Biomarker Levels.

CRP

(n=22)

Inflammation

(n=172)

TNFα

(n=34)
