# Supplementary material for: Cortisol and inflammatory biomarker levels in youths with attention deficit hyperactivity disorder (ADHD): evidence from a systematic review with meta-analysis
Source: Transl Psychiatry. 2021 Aug 19;11:430. doi: 10.1038/s41398-021-01550-0 (PMC8377148; doi:10.1038/s41398-021-01550-0)
Supplement: Supplementary file 3 — Figure S3 [file 41398_2021_1550_MOESM3_ESM.docx]

**a**

**
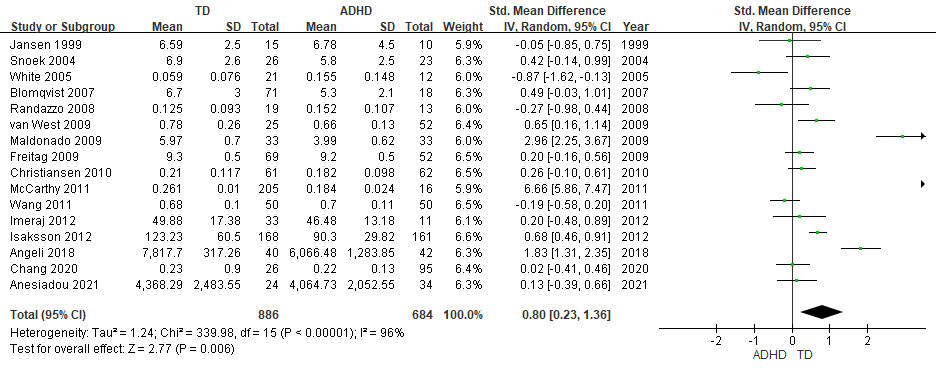
**

**b**

**
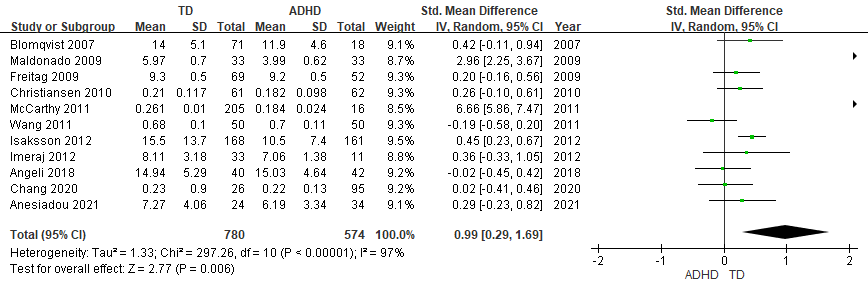
**

**c**


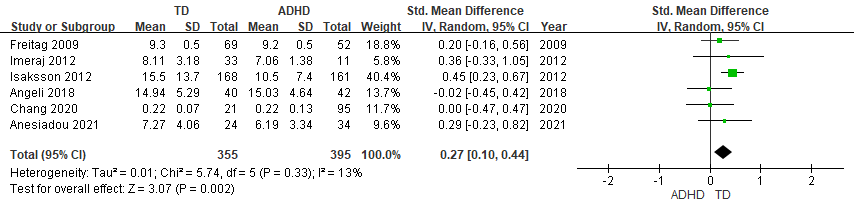


**Supplementary Figure 3. Forest plots comparing effect sizes of (a) salivary cortisol levels, (b) salivary morning cortisol levels and (c) salivary awakening cortisol levels between ADHD and TD groups.**

Forest plots showing effect sizes (Hedges’s g) and 95% confidence intervals (CIs) from individual studies and pooled results comparing **(a)** salivary cortisol levels, **(b)** salivary morning cortisol levels and **(c)** salivary awakening cortisol levels between ADHD population and TD group. ADHD, attention deficit hyperactivity disorder; CI, confidence interval; Std, standard; TD, typically developing youth.
