# Supplementary material for: Cortisol and inflammatory biomarker levels in youths with attention deficit hyperactivity disorder (ADHD): evidence from a systematic review with meta-analysis
Source: Transl Psychiatry. 2021 Aug 19;11:430. doi: 10.1038/s41398-021-01550-0 (PMC8377148; doi:10.1038/s41398-021-01550-0)
Supplement: Supplementary file 5 — Figure S5 [file 41398_2021_1550_MOESM5_ESM.docx]

**a**


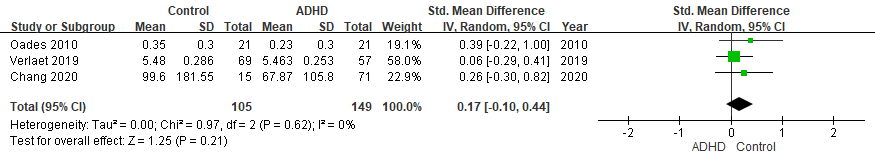


**b**


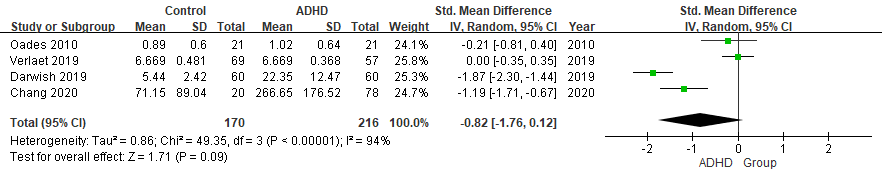


**c**


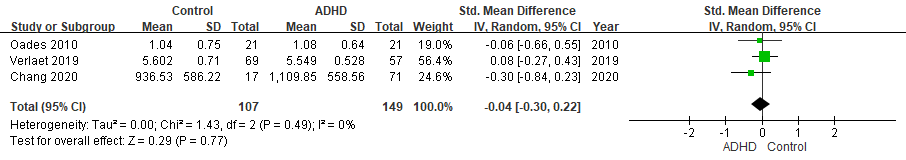


**Supplementary Figure 5. Forest plots comparing effect sizes of IL-1β, (b) IL-6, and (c) IL-10 levels between ADHD and TD groups.**

Forest plots showing effect sizes (Hedges’s g) and 95% confidence intervals (CIs) from individual studies and pooled results comparing (a) IL-1β, (b) IL-6, and (c) IL-10 levels between ADHD population and TD group. ADHD, attention deficit hyperactivity disorder; CI, confidence interval; Std, standard; TD, typically developing youth.
