# Supplementary material for: Cortisol and inflammatory biomarker levels in youths with attention deficit hyperactivity disorder (ADHD): evidence from a systematic review with meta-analysis
Source: Transl Psychiatry. 2021 Aug 19;11:430. doi: 10.1038/s41398-021-01550-0 (PMC8377148; doi:10.1038/s41398-021-01550-0)
Supplement: Supplementary file 6 — Table S1 [file 41398_2021_1550_MOESM6_ESM.docx]

**Supplementary Table S1 Quality Check on the Studies Included in the Meta-Analysis for Cortisol Levels in Children with ADHD**

| **Studies** | **Q1** | **Q2** | **Q3** | **Q4** | **Q5** | **Q6** | **Q7** | **Q8** | **Overall appraisal** |
| --- | --- | --- | --- | --- | --- | --- | --- | --- | --- |
| Jansen (1999)^1^ | Y | Y | Y | Y | Y | Y | Y | Y | I |
| Snoek (2004)^2^ | Y | Y | Y | Y | Y | Y | Y | Y | I |
| White (2005) ^3^ | Y | Y | Y | Y | Y | Y | Y | Y | I |
| Blomqvist (2007)^4^ | Y | Y | Y | Y | Y | Y | Y | Y | I |
| Randazzo(2008)^5^ | Y | Y | Y | Y | Y | Y | Y | Y | I |
| Freitag (2009)^6^ | Y | Y | Y | Y | Y | Y | Y | Y | I |
| Maldonado(2009)^7^ | Y | Y | Y | Y | Y | Y | Y | Y | I |
| Van West (2009)^8^ | Y | Y | Y | Y | Y | Y | Y | Y | I |
| Christiansen(2010)^9^ | Y | Y | Y | Y | Y | Y | Y | Y | I |
| Ma (2011)^10^ | Y | Y | Y | Y | Y | Y | Y | Y | I |
| McCarthy (2011)^11^ | Y | Y | Y | N | Y | Y | Y | Y | I |
| Wang (2011)^12^ | Y | Y | Y | Y | Y | Y | Y | Y | I |
| Imeraj (2012)^13^ | Y | Y | Y | Y | Y | Y | Y | Y | I |
| Isaksson (2012)^14^ | Y | Y | Y | Y | Y | Y | Y | Y | I |
| Kuppili (2017)^15^ | Y | Y | Y | Y | Y | Y | Y | Y | I |
| Angeli (2018)^16^ | Y | Y | Y | Y | Y | Y | Y | Y | I |
| Isik (2018)^17^ | Y | Y | Y | Y | Y | Y | Y | Y | I |
| Anesiadou(2021)^18^ | Y | Y | Y | Y | Y | Y | Y | Y | I |
| Chang (2020)^19^ | Y | Y | Y | Y | Y | Y | Y | Y | I |

Note, JBI, Joanna Briggs Institute; Q1 to Q8 indicates questions based on the JBI Critical Appraisal Checklist for Analytical Cross Sectional Studies; I, included; N, no; NA, not applicable; U, unclear; Y, yes

**1.** Jansen, L.M., Gispen-de Wied, C.C., Jansen, M.A., van der Gaag, R.J., Matthys, W., van Engeland, H. Pituitary-adrenal reactivity in a child psychiatric population: salivary cortisol response to stressors. *Eur Neuropsychopharmacol* 1999;**9**:67-75.

**2.** Snoek, H., Van Goozen, S.H., Matthys, W., Buitelaar, J.K., van Engeland, H. Stress responsivity in children with externalizing behavior disorders. *Dev Psychopathol* 2004;**16**:389-406.

**3.** White, B.P., Mulligan, S.E. Behavioral and physiologic response measures of occupational task performance: a preliminary comparison between typical children and children with attention disorder. *Am J Occup Ther* 2005;**59**:426-436.

**4.** Blomqvist, M., Holmberg, K., Lindblad, F., Fernell, E., Ek, U., Dahllof, G. Salivary cortisol levels and dental anxiety in children with attention deficit hyperactivity disorder. *European J Oral Sci* 2007;**115**:1-6.

**5.** Randazzo, W.T., Dockray, S., Susman, E.J. The stress response in adolescents with inattentive type ADHD symptoms. *Child Psychiatry Hum Dev* 2008;**39**:27-38.

**6.** Freitag, C.M., Hanig, S., Palmason, H., Meyer, J., Wust, S., Seitz, C. Cortisol awakening response in healthy children and children with ADHD: impact of comorbid disorders and psychosocial risk factors. *Psychoneuroendocrinology* 2009;**34**:1019-1028.

**7.** Maldonado, E.F., Trianes, M.V., Cortes, A., Moreno, E., Escobar, M. Salivary cortisol response to a psychosocial stressor on children diagnosed with attention-deficit/hyperactivity disorder: differences between diagnostic subtypes. *Span J Psychol* 2009;**12**:707-714.

**8.** van West, D., Claes, S., Deboutte, D. Differences in hypothalamic-pituitary-adrenal axis functioning among children with ADHD predominantly inattentive and combined types. *Eur Child Adolesc Psychiatry* 2009;**18**:543-553.

**9.** Christiansen, H., Oades, R.D., Psychogiou, L., Hauffa, B.P., Sonuga-Barke, E.J. Does the cortisol response to stress mediate the link between expressed emotion and oppositional behavior in Attention-Deficit/Hyperactivity-Disorder (ADHD)? *Behav Brain Funct*  2010;**6**:45.

**10.** Ma, L., Chen, Y.H., Chen, H., Liu, Y.Y., Wang, Y.X. The function of hypothalamus-pituitary-adrenal axis in children with ADHD. *Brain Res* 2011;**1368**:159-162.

**11.** McCarthy, A.M., Hanrahan, K., Scott, L.M., Zemblidge, N., Kleiber, C., Zimmerman, M.B. Salivary cortisol responsivity to an intravenous catheter insertion in children with attention-deficit/hyperactivity disorder. *J Pediatr Psychol* 2011;**36**:902-910.

**12.** Wang, L.J. et al. Salivary dehydroepiandrosterone, but not cortisol, is associated with attention deficit hyperactivity disorder. *World J Biol Psychiatry* 2011;**12**:99-109.

**13.** Imeraj, L. et al. Time-of-day effects in arousal: disrupted diurnal cortisol profiles in children with ADHD. *J Child Psychol Psychiatry* 2012;**53**:782-789.

**14.** Isaksson J, Nilsson KW, Nyberg F, Hogmark A, Lindblad F. Cortisol levels in children with attention-deficit/hyperactivity disorder. *J Psychiatr Res* 2012;**46**:1398-1405.

**15.** Kuppili, P.P., Pattanayak, R.D., Sagar, R., Mehta, M., Vivekanandhan, S. Thyroid and Cortisol hormones in Attention Deficit Hyperactivity Disorder: A case-control study. *Asian J Psychiatr* Aug;**28**:73-77.

**16.** Angeli, E. et al. Salivary cortisol and alpha-amylase diurnal profiles and stress reactivity in children with Attention Deficit Hyperactivity Disorder. *Psychoneuroendocrinology* 2018;**90**:174-181.

**17.** Isik, U., Bilgic, A., Toker, A., Kilinc, I. Serum levels of cortisol, dehydroepiandrosterone, and oxytocin in children with attention-deficit/hyperactivity disorder combined presentation with and without comorbid conduct disorder. *Psychiatry Res* 2018;**261**:212-219.

**18.** Anesiadou, S. et al. Salivary cortisol and alpha-amylase daily profiles and stress responses to an academic performance test and a moral cognition task in children with neurodevelopmental disorders. *Stress Health* 2021; **37**:45-49.

**19.** Chang, J.P. et al. Cortisol, inflammatory biomarkers and neurotrophins in children and adolescents with attention deficit hyperactivity disorder (ADHD) in Taiwan. *Brain Behav Immun* 2020;**88**:105-113.
