# Supplementary material for: Cortisol and inflammatory biomarker levels in youths with attention deficit hyperactivity disorder (ADHD): evidence from a systematic review with meta-analysis
Source: Transl Psychiatry. 2021 Aug 19;11:430. doi: 10.1038/s41398-021-01550-0 (PMC8377148; doi:10.1038/s41398-021-01550-0)
Supplement: Supplementary file 7 — Table S2 [file 41398_2021_1550_MOESM7_ESM.docx]

**Supplementary Table 2 Quality Check on the Studies Included in the**

**Meta-Analysis for Inflammatory Biomarker Levels in Children with ADHD**

| **Studies** | **Q1** | **Q2** | **Q3** | **Q4** | **Q5** | **Q6** | **Q7** | **Q8** | **Overall appraisal** |
| --- | --- | --- | --- | --- | --- | --- | --- | --- | --- |
| Oades (2010)^1^ | Y | Y | Y | Y | Y | Y | Y | Y | I |
| Verlaet (2019)^2^ | Y | Y | Y | Y | Y | Y | Y | Y | I |
| Darwish (2019)^3^ | Y | Y | Y | Y | Y | Y | Y | Y | I |
| Chang (2020)^4^ | Y | Y | Y | Y | Y | Y | Y | Y | I |

Note, JBI, Joanna Briggs Institute; Q1 to Q8 indicates questions based on the JBI Critical Appraisal Checklist for Analytical Cross Sectional Studies; I, included; N, no; NA, not applicable; U, unclear; Y, yes

**1.** Oades, R.D., Dauvermann, M.R., Schimmelmann, B.G., Schwarz, M.J., Myint, A.M. Attention-deficit hyperactivity disorder (ADHD) and glial integrity: S100B, cytokines and kynurenine metabolism--effects of medication. *Behav Brain Funct*2010;**6**:29.

**2.** Verlaet, A.A.J. et al. Oxidative stress and immune aberrancies in attention-deficit/hyperactivity disorder (ADHD): a case-control comparison. *Eur Child Adolesc Psychiatry*2019;**28**:719-729.

**3.** Darwish, A.H., Elgohary, T.M., Nosair, N.A. Serum Interleukin-6 Level in Children With Attention-Deficit Hyperactivity Disorder (ADHD). *J Child Neurol* 2019;**34**:61-67.

**4.** Chang, J.P. et al. Cortisol, inflammatory biomarkers and neurotrophins in children and adolescents with attention deficit hyperactivity disorder (ADHD) in Taiwan. *Brain Behav Immun* 2020;**88**:105-113.
